# Facile Recrystallization Process for Tuning the Crystal Morphology and Thermal Safety of Industrial Grade PYX

**DOI:** 10.3390/molecules28124735

**Published:** 2023-06-13

**Authors:** Mi Zhang, Jianbo Fu, Hui Ren, Shengfu Li, Xiaole Sun, Qingjie Jiao

**Affiliations:** 1 State Key Laboratory of Explosion Science and Technology, Beijing Institute of Technology, Beijing 100081, China; 15903461635@163.com (M.Z.); bobobo1994@outlook.com (J.F.); jqj@bit.edu.cn (Q.J.); 2 Chongqing Hongyu Precision Industry Group Co., Ltd., Chongqing 402760, Chinasunxiaole060420122@126.com (X.S.)

**Keywords:** PYX, solvent–antisolvent method, morphology, mechanical sensitivity, thermal decomposition

## Abstract

In this study, the crystal appearance of industrial grade 2,6-diamino-3,5-dinitropyridine (PYX) was mostly needle-shaped or rod-shaped with an average aspect ratio of 3.47 and roundness of 0.47. According to national military standards, the explosion percentage of impact sensitivity s about 40% and friction sensitivity is about 60%. To improve loading density and pressing safety, the solvent–antisolvent method was used to optimize the crystal morphology, i.e., to reduce the aspect ratio and increase the roundness value. Firstly, the solubility of PYX in DMSO, DMF, and NMP was measured by the static differential weight method, and the solubility model was established. The results showed that the Apelblat equation and Van’t Hoff equation could be used to clarify the temperature dependence of PYX solubility in a single solvent. Scanning electron microscopy (SEM) was used to characterize the morphology of the recrystallized samples. After recrystallization, the aspect ratio of the samples decreased from 3.47 to 1.19, and roundness increased from 0.47 to 0.86. The morphology was greatly improved, and the particle size decreased. The structures before and after recrystallization were characterized by infrared spectroscopy (IR). The results showed that no chemical structure changes occurred during recrystallization, and the chemical purity was improved by 0.7%. According to the GJB-772A-97 explosion probability method, the mechanical sensitivity of explosives was characterized. After recrystallization, the impact sensitivity of explosives was significantly reduced from 40% to 12%. A differential scanning calorimeter (DSC) was used to study the thermal decomposition. The thermal decomposition temperature peak of the sample after recrystallization was 5 °C higher than that of the raw PYX. The thermal decomposition kinetic parameters of the samples were calculated by AKTS software, and the thermal decomposition process under isothermal conditions was predicted. The results showed that the activation energy (E) of the samples after recrystallization was higher by 37.9~527.6 kJ/mol than raw PYX, so the thermal stability and safety of the recrystallized samples were improved.

## 1. Introduction

Heat-resistant explosive is a kind of explosive which can maintain proper mechanical sensitivity and reliable initiation after enduring a high-temperature environment for a long time and has a high melting point. The research on heat-resistant explosives was first developed to meet military needs, such as spacecraft that need to withstand high-temperature environments, and spacecraft separation at all levels, to meet the special needs of aerospace [1,2]. In the civil field, heat-resistant explosive is mainly used in the perforating blasting equipment of oil and gas wells. 2,2′,4,4′,6,6′-hexanitrostilbene (HNS) [3,4,5,6,7], tetranitro-2,3,5,6-dibenzo-1,3a,4,6a-tetraazapentalene (TACOT) [8,9,10,11], 2,2′,2″,4,4′,4″,6,6′,6″-nonanitroterphenyl (NONA) [12,13], 1,3,5-triamino-2,4,6-trinitrobenzene (TATB) [14,15,16,17,18,19], and 2,6-bis(picrylamino)-3,5-dinitropyridine (PYX) [20,21,22,23,24,25] are commonly used in heat-resistant explosives. The energy level of TACOT is low and the synthesis steps are complex. NONA is superior to PYX in overall performance, but the separation process is too complex to be mass produced and is only used in impact plate detonators. TATB has a small particle size, poor press formability, and high cost. PYX is a heat-resistant energetic material developed by Los Alamos National Laboratory in the 1960s. The synthesis process of PYX is simple, and its thermal stability and energy level are better than HNS. The pyridine ring in the PYX molecule enhances its overall conjugacy, and the imino group connecting the pyridine ring and benzene ring enables easy formation of intramolecular and intermolecular hydrogen bonds with the adjacent nitro group, which helps to improve the thermal stability of the compound [23]. The thermal decomposition peak temperature of PYX is 370 °C, and the thermal stability is well above 350 °C. It has a high melting point, no crystallization, sublimation, and decomposition in a wide range of temperatures, and has good physical and chemical stability [25]. In addition, PYX has good low-temperature resistance, radiation resistance, and electrostatic spark resistance. In America, the engineering preparation of PYX has been realized several decades ago, and now our country also has achieved an annual production tonnage scale. However, the crystal morphology of PYX is mostly needle-like or rod-like crystal, with low packing density and poor dispersion, which makes it difficult to meet the requirements of modern weapons charging technology. At present, PYX is mainly used in petroleum perforating charges and has not been widely used in the military field. Therefore, it is very important to improve the crystal morphology of PYX for its further application in the military industry.

Solution recrystallization plays an important role not only in the separation and purification of explosives, but also in the control of particle size and morphology of explosive crystals [26,27]. Recrystallization in solution is one of the most commonly used crystal means of explosives, including the cooling method, solvent–antisolvent method, evaporation method, etc. The solvent–antisolvent recrystallization method has the advantages of simple principle, mild operating conditions, less energy consumption, low cost, and it is easy to scale up. It is widely used in the crystallization of explosives [28,29,30,31,32,33]. The solvent–antisolvent method is based on the principle of different solubility of the solute in different solvents; a certain concentration of solution is added to a certain amount of antisolvent, so that the solute is oversaturated and precipitated. The function of antisolvent is to reduce the solubility of the solute in the solution and promote crystal precipitation. Its physical and chemical properties directly affect the mixing rate of solute and solution, thus affecting the nucleation and growth of crystals in solution and further affecting the final morphology of crystals. Therefore, it is possible to control the crystal morphology of explosives by changing the experimental parameters of antisolvent crystallization. At present, the reports on PYX mostly focus on synthesis process optimization, and there are few studies on recrystallization.

In this paper, the solubility of PYX in DMSO, DMF, and NMP was measured by the static differential weight method. The recrystallization of PYX was carried out by the solvent–antisolvent method. The crystal morphology, structure, thermal properties, and mechanical sensitivity of PYX before and after recrystallization were compared and analyzed, which provided the basis for crystal improvement, solvent and antisolvent selection, and recrystallization process optimization of PYX.

## 2. Results and Discussion

### 2.1. Solubility Model

**Figure 1:**
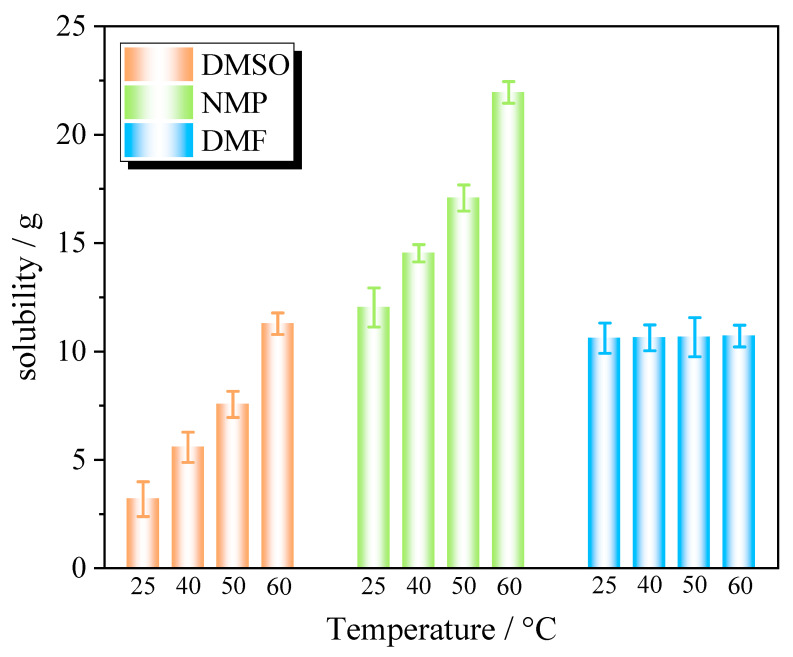
The histogram of PYX solubility.

The solubility of PYX in DMSO, DMF, and NMP variations with temperature are shown in Figure 1. PYX has the highest solubility in NMP, followed by DMF, and the lowest solubility in DMSO. The solubility varies greatly with temprature in NMP and increases with temperature. The solubility also increases with the increase in temperature in DMSO. The influence of temperature on the solubility of DMF is not obvious. Therefore, when using the solvent–antisolvent recrystallization method, solvents with higher solubility, namely DMF and NMP, were selected. In the process of cooling recrystallization, NMP and DMSO are preferred as solvents.

The empirical model Apelblat equation and Van’t Hoff equation were used to correlate the solubility of PYX in pure solvents at different temperatures. Apelblat assumed that the enthalpy of the solution was a linear function of temperature, and derived the solubility Equation (1) from the Clausius–Clapeyron equation:

(1) $${\mathrm{lnx}}_{A}=A+\frac{B}{T}+\mathrm{ClnT}$$

where x_A_ is the molar fraction solubility of the solute, T is the absolute temperature, and A, B, and C are all model parameters.

For ideal solutions, solubility can be predicted by the Van’t Hoff equation, where x_A_ represents the molar fraction solubility of the solute, T_f_ represents the melting temperature of the solute, ΔH_f_ and ΔS_f_ represent the molar enthalpy (J·mol^−1^) and molar entropy (J·mol^−1^) of the solute, respectively. R is the molar gas constant (8.314 J·mol^−1^·K^−1^).

(2) $${\mathrm{lnx}}_{A}=\frac{{\Delta H}_{f}}{R}[\frac{1}{T_{f}}-\frac{1}{T}]=-\frac{{\Delta H}_{f}}{\mathrm{RT}}+\frac{{\Delta S}_{f}}{R}$$

In practice, since there is no ideal solution, the interaction between solute and solvent is often not negligible. Therefore, for non-ideal solutions, the enthalpy of dissolution ΔH_d_ is used instead of ΔH_f_, ΔS_d_ instead of ΔS_f_, and the enthalpy and the entropy of mixing are taken into account. The Van’t Hoff equation changes to Equation (3).

(3) $${\mathrm{lnx}}_{A}=\frac{-{\Delta H}_{d}}{\mathrm{RT}}+\frac{{\Delta S}_{f}}{R}$$

The equation can be further simplified as in Equation (4),

(4) $${\mathrm{lnx}}_{A}=\frac{a}{T}+b$$

where both a and b are model parameters.

The applicability evaluation of the above model is measured by the squared correlation coefficient R^2^, the relative deviation RD and the root-mean-square deviation RMSD. The definitions are shown in Equations (5) and (6), where x_exp_ represents the solubility value measured in the experiment and x_cal_ represents the calculated value of the solubility.

(5) $$\mathrm{RD}=\frac{x_{\exp}-x_{\mathrm{cal}}}{x_{\exp}}\times 100\%$$

(6) $$\mathrm{RMSD}={\left[\frac{1}{N}\overset{N}{\underset{i=1}{\sum }}{{(x}_{\exp}-x_{\mathrm{cal}})}^{2}\right]}^{1/2}$$

The Abelbalt equation and Van’t Hoff equation regression model parameters of PYX in different solvents are shown in Table 1 and Table 2, and the relative deviation and root-mean-square deviation between measured and calculated solubility values are also listed in the table.

The squared correlation coefficient R^2^ of the fitting results by the Apelblat and Van’t Hoff model is greater than 0.99, and the root-mean-square deviation RMSD is less than 3%, indicating that the two models have good fitting results. Two solubility models, the Apelblat equation and Van’t Hoff equation, were used to predict the solubility and compared with the experimental values. The results are shown in Table 3. The results show that the Apelblat equation and Van’t Hoff equation can describe the solubility of PYX in different solvents compatibly.

### 2.2. Morphology and Structure

**Figure 2:**
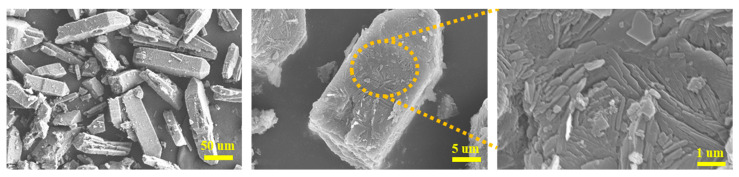
The SEM image of raw PYX.

The morphology of raw PYX was characterized by SEM. The microstructure under different magnifications was collected, and the results are shown in Figure 2. The morphology of the raw PYX is generally rod-like, with a length of about 100~200 um and a length–diameter ratio of about 5~10. In addition, a large number of broken and small crystals (less than 100 um in length) can also be observed, and the homogeneity of the material is poor. The crystal surface is uneven, and a large number of fragments are attached.

**Figure 3:**
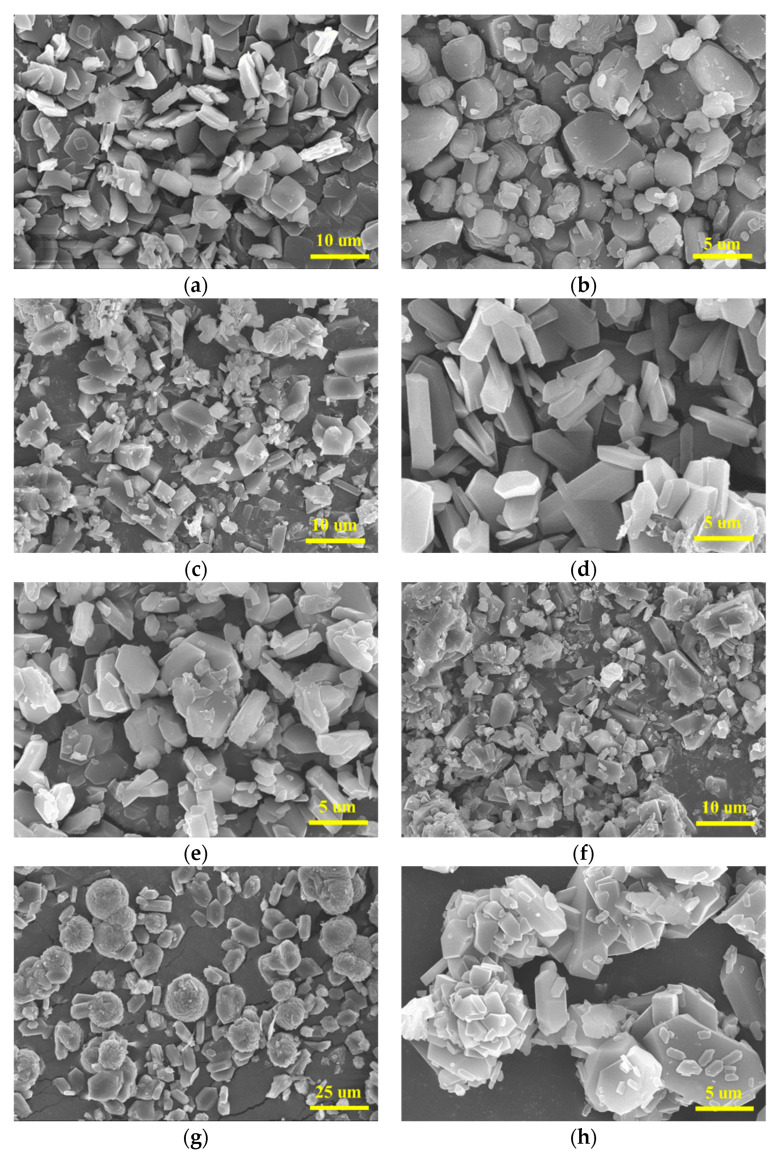
Recrystallization diagram of PYX in different solvent systems. (**a**) NMP—ethanol. (**b**) NMP—*n*-hexane. (**c**) NMP—acetonitrile. (**d**) NMP—ethyl acetate. (**e**) NMP—dichloromethane. (**f**) DMF—acetonitrile. (**g**) DMF—ethanol. (**h**) DMF—dichloromethane.

PYX was recrystallized with different solvent–antisolvent systems under the same recrystallization condition (recrystallization temperature 40 °C. Rapid addition volume is one time that of solvent. Antisolvent droplet acceleration rate 4 mL/min. Solvent–antisolvent ratio 1:7. Stirring rate 400 r/min, the stirring method was magnetic agitation). Figure 3 shows the recrystallization scanning electron microscope images of different solvent–antisolvent systems. PYX in an NMP–anhydrous ethanol system presents a square sheet structure with a particle size of about 5–10 um. In the NMP–*n*-hexane system, it shows a cube with smooth corners and a particle size of about 5 um. The crystal growth is chaotic and amorphous in NMP–acetonitrile. In the NMP–ethyl acetate system, the crystal is rod-like, and the crystal surface is smoother than that of the raw material. In the systems of NMP–dichloromethane, DMF–acetonitrile, and DMF–dichloromethane, amorphous crystal distribution and a large number of small granular crystals exist. In a DMF–anhydrous ethanol system, the crystal appears spherical, but the crystal surface is not smooth, similar to a sawtooth. The main factor affecting crystal morphology under the same crystallization conditions is the performance of the solvent. The interaction between solvent and solute not only affects the solubility of the solution, but also has a great influence on crystal growth morphology. This may be due to the strong adsorption between solvent molecules and solute molecules on the crystal surface, which affects the growth rate of each crystal surface and thus affects the crystal growth morphology. In addition, the crystal growth environment will change with different polarities of the solvent, and the crystal shape of the final product will also change. Therefore, the crystal morphology of PYX is different in different solvent systems.

**Figure 4:**
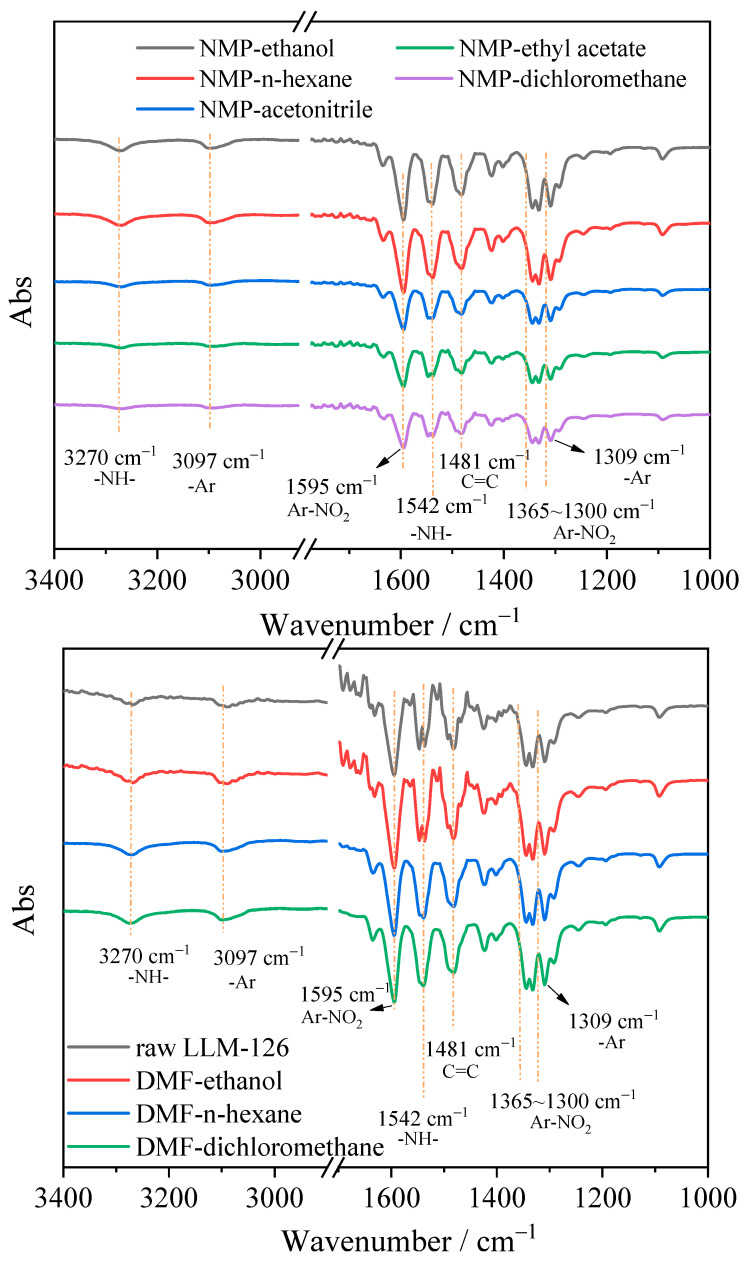
The IR spectra of raw and recrystallized PYX.

The chemical interaction between the solute and the solvent, and whether the solute will undergo structural changes in the solvent, are the main considerations in the recrystallization process. The infrared spectra of recrystallization of different solvent systems were tested, and the results are shown in Figure 4. The characteristic peaks of 3270 cm^−1^ and 1542 cm^−1^ are the shear bending vibration of imino (-NH-). The characteristic peak at 3097 cm^−1^ is the characteristic peak of the aromatic ring (-Ar), including the benzene ring and pyridine ring. The characteristic peaks at 1595 cm^−1^ and 1300~1365 cm^−1^ correspond to the stretching vibration of nitro (-NO_2_), and the characteristic peak at 1481 cm^−1^ is C=C in the benzene ring. The infrared characteristic peaks of samples of all solvent systems correspond to those of raw PYX, and no functional group changes occur, so chemical structure changes do not occur in the process of recrystallization.

### 2.3. Crystal Morphology and Particle Size Distribution

At the same solid content, the higher the degree of sphericity of crystal particles, and the smoother the surface, the easier the slip between particles becomes. Meanwhile, spherical particles have the smallest specific surface area and are more likely to be wetted by liquid phase components. To obtain good rheological properties of the mixed explosive formula, by reducing the viscosity of the formula system and the difficulty of the pouring process in the processing process, and by improving the solid content of the single-compound explosive, the energy density, and detonation performance of the mixed explosive, it is required that the energetic crystal particles should be as close to spherical as possible.

To evaluate the crystal morphology of explosives, the aspect ratio of the minimum outer rectangle of the crystal particle (Ψ_ratio_) and the particle roundness value (Φ_circularity_) as the shape factor, to characterize the shape of the particle quantitatively, were measured [34]. The calculation formula is shown in Equations (7) and (8).

(7) $$\Psi_{\mathrm{ratio}}=\frac{L_{\mathrm{length}}}{W_{\mathrm{width}}}$$

(8) $$\Phi_{\mathrm{circularity}}=\frac{4\pi \times A}{P_{\mathrm{bordeiline}}^{2}}$$

where L_length_ is the length of the minimum enclosing rectangle, W_width_ is the width of the minimum enclosing rectangle, A is the projected area of the particle, and P_borderline_ is the perimeter of the projection.

**Figure 5:**
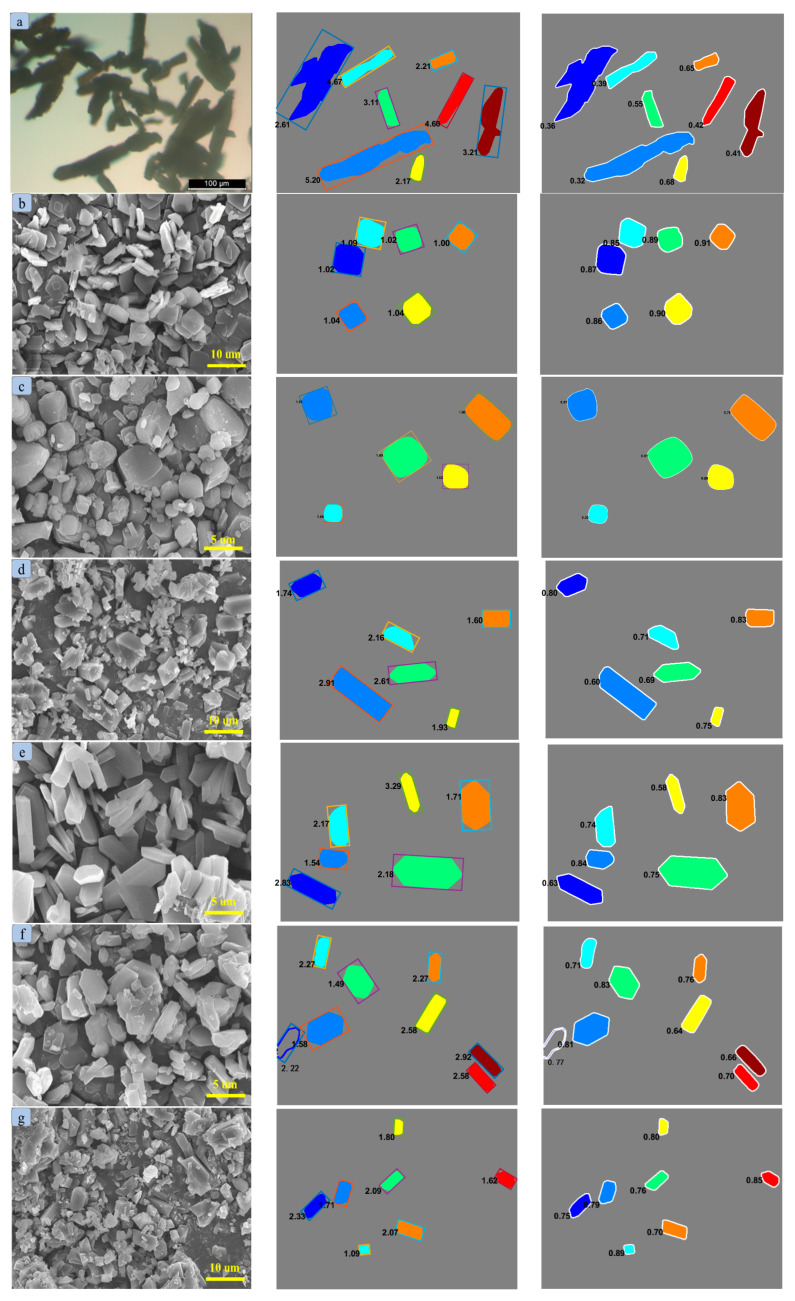

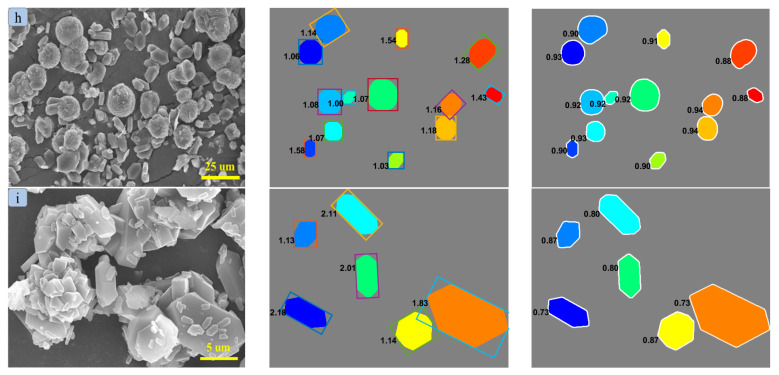
The images of aspect ratio and roundness value of raw PYX (**a**) and recrystallized PYX (**b**–**i**).

**Figure 6:**
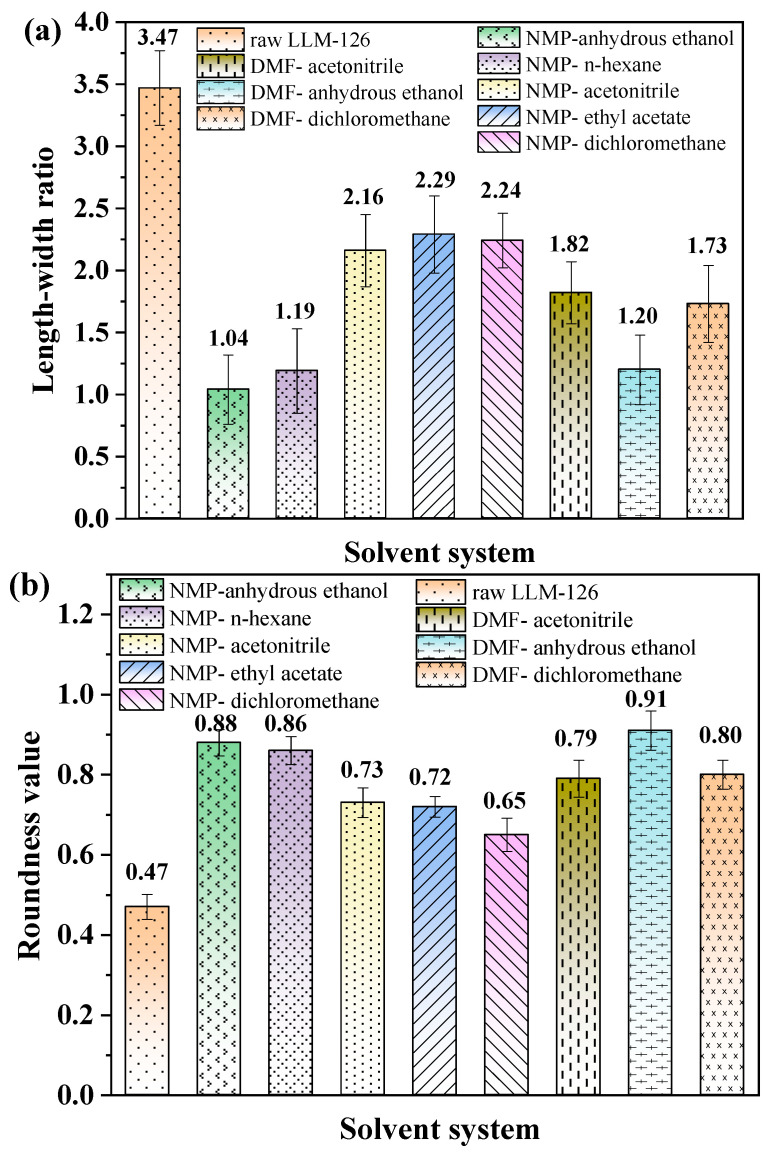
The histogram of aspect ratio (**a**) and roundness value (**b**) of raw PYX and recrystallized PYX.

The aspect ratio and roundness are recognized by MATLAB. Five pictures were taken of each solvent system and the average was calculated; the results are shown in Figure 5 and Figure 6. The raw PYX used for recrystallization is mostly “long needle-shape”, and its average aspect ratio and roundness values were about 3.47 and 0.47. After recrystallization by different processes, the aspect ratio and roundness value of the crystals are improved greatly. The aspect ratio was reduced to 1.04, 1.19, and 1.20 of recrystallized PYX in the systems of NMP–ethanol, NMP–*n*-hexane, and NMP–ethanol, respectively. The roundness value was increased to 0.88, 0.86, and 0.91. When anhydrous ethanol was used as an antisolvent, NMP and DMF were used as solvents, and the aspect ratio and roundness value of the crystal were improved significantly. Proper stirring can enhance the fluidity of solute in liquid, promote the mixing of solvent and antisolvent, and avoid the “explosive nucleation” caused by high local desaturation. At the same time, according to the diffusion principle of crystal growth, the stirring promotes the crystal to move into the mother solution, thus reducing the difference between the crystal interface layer (or adsorption layer) concentration and the concentration of the bulk solution. The decrease in concentration difference weakens the difference in growth rate among crystal faces, so that all crystal faces can grow synchronously at similar growth rates as much as possible, thus obtaining polyhedral spherical crystals.

During the recrystallization process, the introduction of solvent composites or impurities will adversely affect the stability and application prospect of PYX. Chemical purity is an important technical index of energetic compounds. High-performance liquid chromatography (HPLC) is generally used to detect the purity of energetic composites. The purity of PYX was calculated according to the peak area normalization. The purities of raw PYX and recrystallized PYX in the NMP–*n*-hexane system were 98.89% and 99.37%.

**Figure 7:**
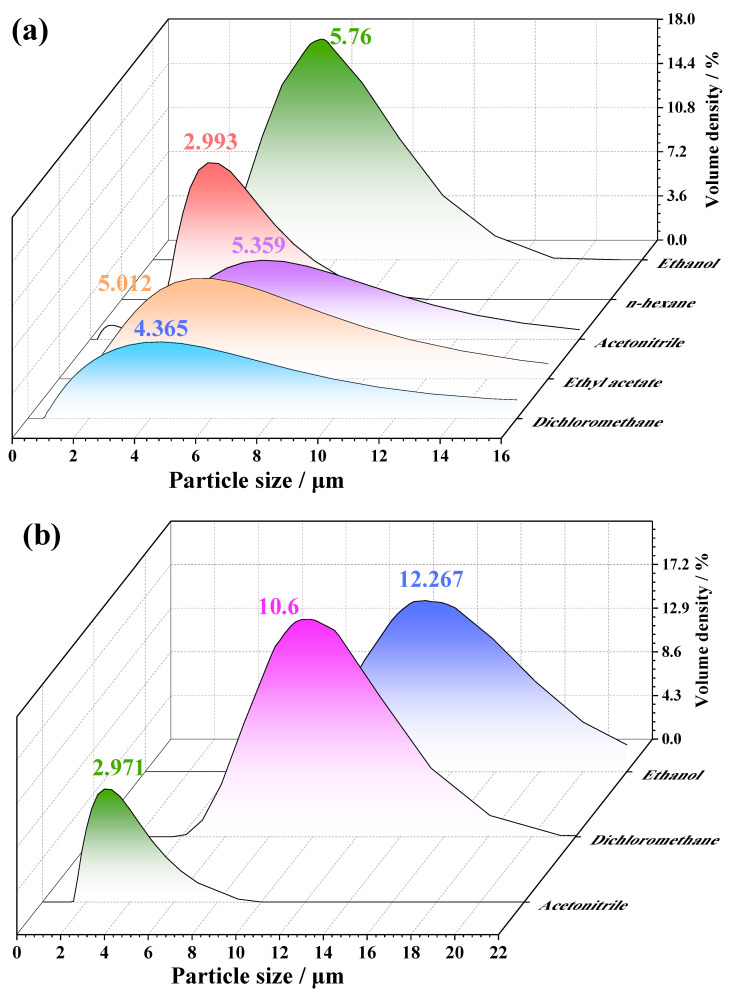
The particle size distribution diagram of recrystallized PYX (**a**) NMP solvent system, (**b**) DMF solvent system.

The particle size distribution of recrystallized samples was characterized by a laser particle size analyzer. The results are shown in Figure 7. The median diameter of PYX is 73.751. After recrystallization, the grain size of PYX decreases obviously. The crystal size of the solvent–antisolvent system is almost less than 10 μm. The rapid addition of antisolvent in the early stage of crystallization leads to the acceleration of the nucleation rate, which is conducive to the formation of small grains. High-speed stirring can promote the formation of crystal nuclei and is not conducive to the adhesion and growth of solute molecules on the crystal surface. Moreover, the collision between crystals is increased, resulting in the decrease in crystal size.

### 2.4. Mechanical Sensitivity

**Figure 8:**
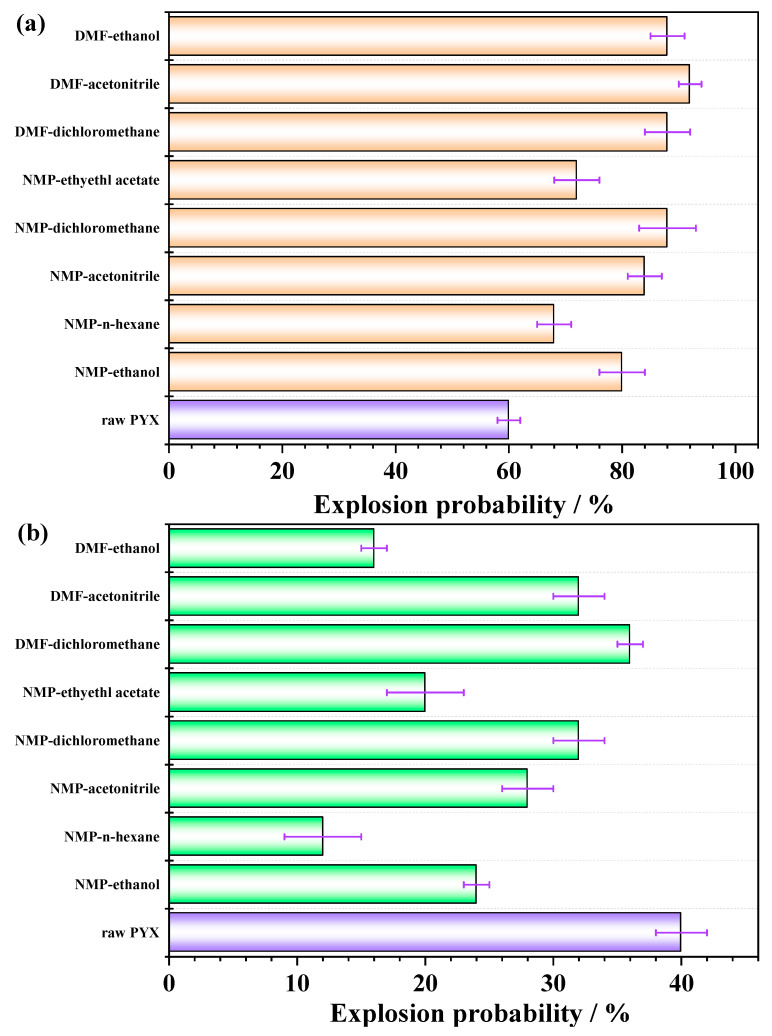
The friction sensitivity (**a**) and impact sensitivity (**b**) of PYX.

Sensitivity refers to the degree of difficulty for energetic materials to explode under external stimuli (heat, impact, impact, laser, etc.), which is one of the most important indexes to evaluate the safety performance of explosives. The sensitivity will directly affect the synthesis, transportation, storage, and use of energetic materials. Friction sensitivity and impact sensitivity of samples before and after recrystallization were tested, and the results are shown in Figure 8. The impact sensitivity of PYX is 40%, and the friction sensitivity is 60%. After recrystallization, the impact sensitivity of the samples decreased to 20%, 12%, 24%, 32%, 28%, 36%, 32%, and 16%, respectively. The friction sensitivity was increased to 72%, 68%, 80%, 88%, 84%, 88%, 92%, and 88% after recrystallization, respectively.

Under mechanical action, mechanical energy is first transformed into heat energy. The heat generated has no time to evenly distribute on the whole sample, but concentrates on individual small points in the sample to form a “hot spot”. Heat is diffused around the hot spot in the form of deflagration, and then from combustion to low-speed explosion, until stable detonation. The size and porosity of the holes between the explosive particles are the main factors that can form the “hot spot” on some small points in the explosive. When the explosive density is constant, the size of the holes depends on the size of the explosive particles. The smaller the explosive particles are, the smaller the size of the holes that will be formed, and the smaller the hot spot size will be formed during adiabatic compression, so it is not easy to detonate and the sensitivity is low. Small-size explosive has a large specific surface area and high surface energy, the force borne by the unit surface decreases, and the thermal conductivity of particles increases. In addition, the size of microbubbles inside the crystal will be smaller with the decrease in crystal size. The reduction of microbubble size will result in less heat release from the adiabatic compression of explosives under external impact and more difficult hot spot formation. Therefore, the impact sensitivity of PYX decreases after recrystallization. The hot spot formed by friction is mainly caused by local friction, plastic deformation, and the viscous flow of explosives at the protruding point of the explosive crystal. As the particle size of the explosive decreases, the relative flow velocity between particles increases, thus the viscosity coefficient increases and the viscous flow generates more heat. Therefore, the friction sensitivity of PYX after recrystallization is higher than that of the raw material. In addition, there are broken crystals after recrystallization, and the particle size becomes smaller after solvent–antisolvent crystallization. The impact sensitivity sample is loosely placed in the mold, and the friction sensitivity sample needs to be pressurized, thereby increasing the friction between particles.

### 2.5. Thermal Decomposition

**Figure 9:**
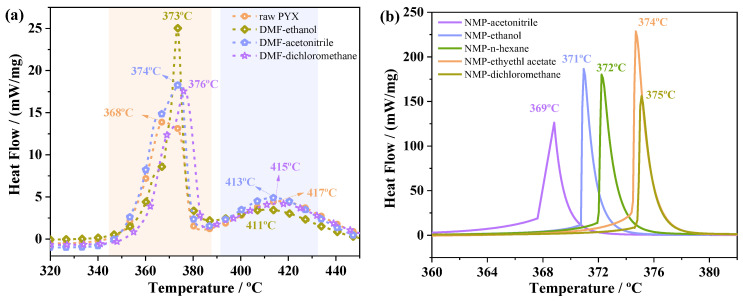
The DSC curve of raw PYX and recrystallization sample at DMF solvent. system (**a**) and NMP solvent system (**b**).

Thermal stability is an important test index of heat-resistant explosives. The thermal decomposition behavior of PYX before and after recrystallization was studied by DSC. Based on DSC data, the thermal decomposition kinetic parameters of PYX were calculated by AKTS software, and the thermal decomposition process of PYX was predicted under isothermal conditions. Figure 9 shows the DSC curves after recrystallization in different solvent systems. The thermal decomposition of PYX and DMF solvent system recrystallization samples is mainly divided into two stages. The main decomposition peak temperature of raw PYX is 368 °C, and the second stage decomposition peak temperature is 417 °C. With DMF as the solvent and ethanol, acetonitrile, and methylene chloride as the antisolvent, the first thermal decomposition temperatures peaks of the samples after recrystallization were 373 °C, 374 °C, and 376 °C, respectively. The peak temperatures in the second stage were 413 °C, 411 °C, and 415 °C, respectively. The peak temperatures of decomposition in the first stage were delayed by 5 °C, 6 °C, and 8 °C respectively, but the peak temperatures in the second stage were advanced by about 4 °C, 6 °C and 2 °C respectively. With NMP as solvent and acetonitrile, ethanol, *n*-hexane, ethyl acetate, and dichloromethane as an inverse solvent, the thermal decomposition temperatures of the samples after recrystallization were 369 °C, 371 °C, 372 °C, 374 °C, and 375 °C, respectively. The peak temperatures were delayed by about 1 °C, 3 °C, 4 °C, 6 °C, and 7 °C compared with raw materials, respectively. The second stage decomposition of recrystallized samples in NMP solvent system is not obvious. In conclusion, the main decomposition peak temperatures of samples after recrystallization were all delayed, indicating that recrystallization did not affect the thermal stability of PYX, but could improve it.

AKTS software is mainly used for reaction kinetics analysis of various types of thermal analysis data (DSC, DTA, TGA, TG-MS, or TG-FTIR) of raw materials and products in the research and development process. The software mainly includes two parts: thermal dynamics and thermal safety. Based on the Friedman Method [35] (Equation (9)), the Ozawa Method [36] (Equation (10)), and the Kissinger Technique [37] (Equation (11)), the thermodynamics module mainly studies the variation trend of the material reaction process (α) and reaction rate (dα/dt) with temperature (T), and the variation of activation energy (E) with α,

(9) $$\ln\left[\beta \frac{d_{\alpha }}{d_{T}}\right]{=\mathrm{lnAe}}^{-\frac{E}{\mathrm{RT}}}f(\alpha )$$

(10) $$\mathrm{lg}\beta =\mathrm{lg}\left[\frac{\mathrm{AE}}{\mathrm{RG}(\alpha )}\right]-2.305-0.4567\frac{E}{{\mathrm{RT}}_{p}}$$

(11) $$\ln\frac{\beta }{T_{p}^{2}}=\ln\frac{\mathrm{RA}}{E}-\frac{E}{{\mathrm{RT}}_{p}}$$

where β is the different heating rate, °C·min^−1^; α is the conversion rate; T is the reaction temperature, K; A is the pre-exponential factor, s^−1^; E is the activation energy, kJ·mol^−1^; R is the gas constant, J·mol^−1^·K^−1^; f(α) is the reaction mechanism function; and T_p_ is peak temperature, K.

In the Friedman method, f(α) is constant for any fixed α, 1/T is used as the abscissa, and $\ln\left[\frac{d_{\alpha }}{d_{T}}\right]$ is the ordinate, and it is fitted by the least squares method. The E is obtained from the slope, and A is obtained from the intercept. The value of f(α) cannot be obtained by this method; only the product of A and f(α) can be obtained. Although the value of f(α) cannot be obtained by this method, many assumptions in the process of reaction can be avoided, making the calculation results more universal [38]. The Ozawa method requires a series of experiments with different heating rates. Since the α values at the peak temperature T_p_ of each thermal spectrum are approximately equal under different β, the E values can be determined by the linear relationship of lgβ − 1/T. This method avoids the choice of reaction mechanism function and directly calculates the E value. Compared with other methods, it avoids the error which may be caused by different assumptions of the reaction mechanism function. The Kissinger method uses $\ln\left[\frac{\beta }{T_{p}^{2}}\right]$ as ordinate, 1/T_p_ as abscissa, and fitting to obtain a straight line. E is calculated by slope and A is obtained by intercept. The calculated activation energy is a fixed value.

The thermal decomposition kinetics of PYX was studied by AKTS software. The reaction process and reaction rate of raw PYX were compared with that of PYX in the NMP–*n*-hexane solvent system. The change in activation energy of PYX in the main decomposition stage with reactivity before and after recrystallization was studied.

**Figure 10:**
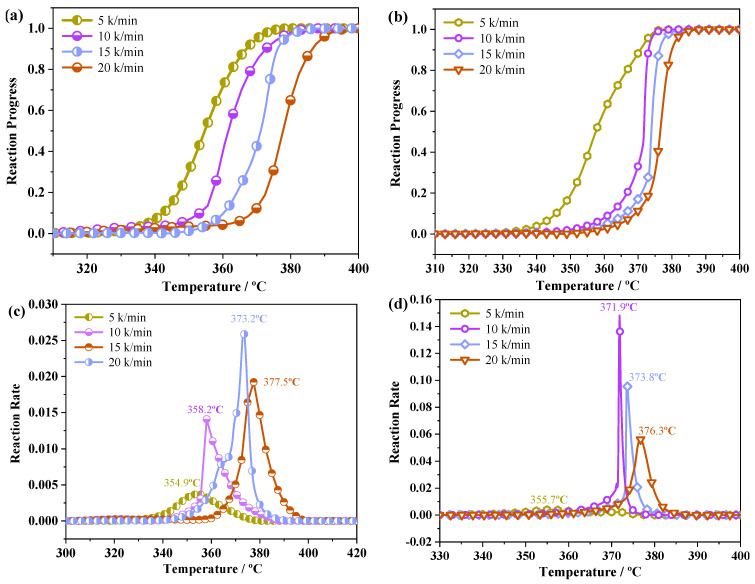
The temperature–reaction progress (**a**,**b**) and temperature–reaction rate (**c**,**d**) curve of raw PYX and recrystallization sample.

Figure 10 shows the reaction process and reaction rate curves of raw PYX and recrystallization sample (NMP–*n*-hexane) at different heating rates as a function of temperature. The temperatures of raw PYX at different heating rates to reach the maximum reaction rate were 355 °C, 358 °C, 373 °C, and 378 °C, respectively. After recrystallization, the temperature that the samples reached the maximum reaction rate were 356 °C, 372 °C, 374 °C, and 376 °C, respectively. The temperature that the sample after recrystallization reached the maximum reaction rate was later than that of raw PYX. It indicates that the thermal stability of the sample is improved after recrystallization. At different heating rates, the samples showed the same reaction trend before and after recrystallization, and the reaction rate first increased to a certain peak and then decreased. The results showed that recrystallization did not change the thermal decomposition mechanism of the main decomposition of materials, and the thermal decomposition mechanism was not affected by the heating rate.

**Figure 11:**
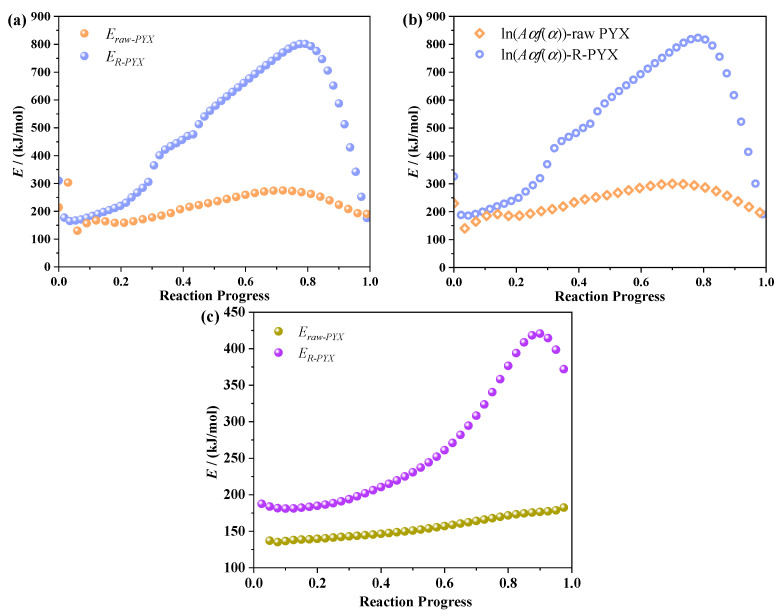
The variation curve of E and ln(A_α_f(α)) with reaction progress of Friedman (**a**,**b**) and Ozawa (**c**).

Figure 11 is the change curve of E and ln(A_α_f(α)) with the reaction progress of raw PYX and recrystallization sample (R-PYX) by the Friedman method and Ozawa method. The activation energies of PYX and recrystallized samples by the Friedman method are 129.8~276.3 kJ/mol and 167.7~803.9 kJ/mol, respectively. The E changes gently at α = 0.1~0.3. When α = 0.3~0.78, the activation energy increased rapidly. α = 0.78~1, activation energy decreased rapidly. The maximum activation energy of PYX was 276.3 kJ/mol when the reaction process was 0.7. The maximum activation energy of the recrystallized sample was 809.3kJ/mol when the reaction process was 0.78. The activation energies of PYX and recrystallized samples calculated by the Ozawa method were 135.5~184.1 kJ/mol and 183.6~420.9 kJ/mol, respectively. The variation trend of the activation energy of the samples after recrystallization is consistent with that calculated by the Friedman method, and the maximum activation energy (420.9 kJ/mol) was reached at α = 0.89. The activation energy of raw PYX increased with the increase in reactivity during the whole decomposition process until the end of the reaction, and the maximum activation energy was 184.1 kJ/mol. The values of E calculated by the Kissinger method were 136.8 kJ/mol and 214.2 kJ/mol, respectively. Activation energy refers to the amount of energy required for a molecule to change from a normal to an active state prone to chemical reactions. The rate of the chemical reaction is closely related to the value of its activation energy. The higher activation energy with the slower reaction rate. After recrystallization, the α of the material to reach the maximum activation energy is about 8% later than that of the raw material, and the maximum activation energy is 2.9 times of the raw PYX. Therefore, the recrystallization PYX decomposition reaction is more difficult to occur, and the thermal stability is better than raw PYX.

**Figure 12:**
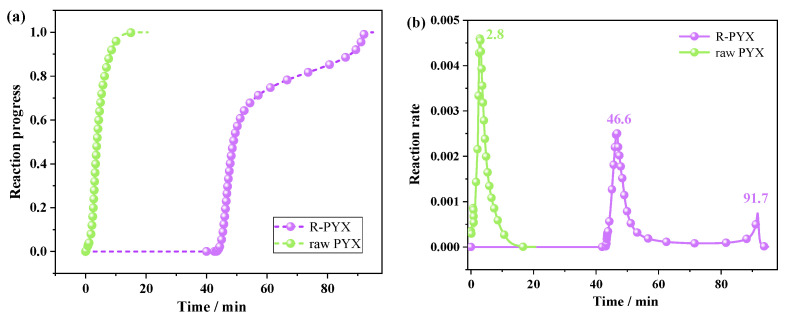
AKTS software predicts the reaction process (**a**) and reaction rate (**b**) curve with time under constant temperature conditions 350 °C of raw PYX and recrystallization sample.

Based on conventional thermal tests, AKTS can infer the properties and behaviors of the substance, give kinetic parameters, and predict the reaction process under various temperatures and conditions. It is possible to predict reaction processes in complex temperature distribution situations, such as isothermal, non-isothermal, stepwise heating, periodic temperature changes, etc. PYX is a heat-resistant explosive, its thermal safety under constant temperature conditions is also an important index to measure its heat resistance. AKTS software was used to predict the reaction process of PYX and recrystallization samples at a constant temperature of 350 °C with time, and the results were shown in Figure 12. The decomposition of raw PYX directly began under the constant temperature of 350 °C, the maximum reaction rate was reached in 2.8 min, and the decomposition was completed at 16.6 min. After recrystallization, the sample was stored at 350 °C for 42.8 min and began to decompose, reaching the maximum decomposition rate at 46.6 min. The maximum decomposition rate of the second stage was at 91.7 min, and then completely decomposed at 95.2 min. Therefore, the thermal stability of the recrystallized sample is better than that of raw PYX.

## 3. Experimental

### 3.1. Materials

PYX was provided by Shanxi Beihua Guanlv Chemical Industry Co., Ltd. (Yongji City, China). N-Methyl pyrrolidone (NMP), N,N-Dimethylformamide (DMF), Dimethyl sulfoxide (DMSO), Ethanol absolute, Hexane, acetonitrile, ethyl acetate, and dichloromethane (purity of 99.9%) were provided by Beijing Tongguang Fine Chemical Co., Ltd. (Beijing city, China). The following were also used: electronic balance (XPR204S/AC, METTLER TOLEDO Co., Ltd., Zurich, Switzerland); oil bath pan (DF-101S, Shanghai Lichen Technology Instrument Co., Ltd., Shanghai, China); peristaltic pump (BT100S, Baoding LEADFLUID Co., Ltd., Baoding, China); digital display overhead mechanical agitator (OS20-S, Zhejiang Jianran Instrument Equipment Co., Ltd., Shaoxing, China).

### 3.2. Methods

**Figure 13:**
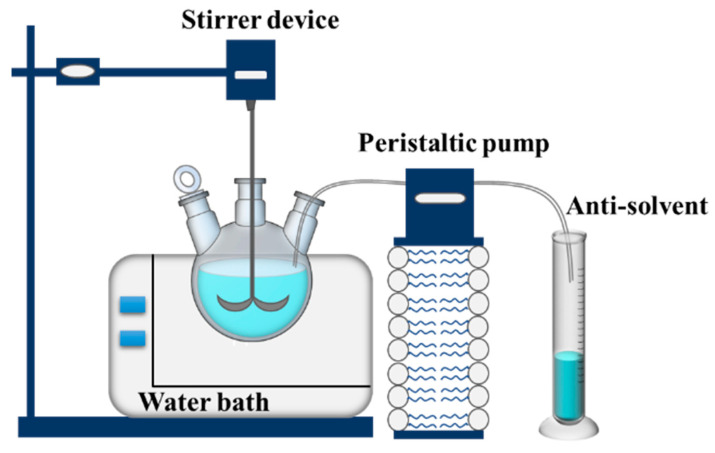
The schematic diagram of the recrystallization device.

The saturated solution was first prepared according to the solubility data. The water bath was turned on and the temperature adjusted to 40 °C, and then the mechanical stirring motor was turned on and stirred slowly until the solute was completely dissolved. The peristaltic pump was turned on and the antisolvent added to the three-neck flask at a fixed rate. After the antisolvent was added, the solution was filtered and the residual solvent was washed with anhydrous ethanol. After vacuum filtration of the crystal slurry, the filtered crystal was dried in the oven to obtain the sample. Figure 13 is the schematic diagram of the recrystallization device.

The reference shows that PYX is soluble in DMSO, DMF, and NMP, so the three solvents were selected for the study [22,23,24,25]. Ka et al. [39] summarized seven aspects of solvent selection during the crystallization process: (1) the chemical interaction between solute and solvent, (2) the boiling point of the solvent and the thermodynamic properties of the solute according to the operating temperature, (3) whether there is a higher yield in the selected solvent, (4) whether the structure of the solute changes in the solvent, (5) whether the solvent is conducive to recovery, (6) whether the solvent has an impact on the environment and operator health, and (7) solubility curve type and operating temperature elastic range. The International Conference on Harmonization (ICH) divides solvents into three categories according to their impact on the environment. The first category refers to substances that have a very large impact on the environment and are likely to cause cancer, such as benzene; the second type of solvent has certain toxic substances, such as methanol; the third type of solvent has very little impact on the environment, such as ethanol or 2-propyl alcohol. Usually, the solvents used in the crystallization process are the second and third kinds of solvents. Solvents are usually divided into aromatic hydrocarbon, alicyclic hydrocarbon, halogenated hydrocarbon, alcohols, ethers, esters, ketones, and other categories. Toluene is flammable, vapor and air can form an explosive mixture, and a mixture volume concentration in a low range can explode. Acetone vapor can form an explosive mixture with air. In case of an open fire and high heat, it is easy to ignite and explode and generate harmful products with toxicity. At the same time, PYX is slightly soluble in acetone, which affects the yield. Therefore, ethyl acetate, *n*-hexane, anhydrous ethanol, dichloromethane, and acetonitrile were selected as the reverse solvents for recrystallization process research.

The solubility of PYX in DMSO, DMF, and NMP was measured by the static differential weight method. The principle is to place excessive solid solute in a liquid solvent, sealed and heated to a certain temperature, then constant temperature stirring to promote the solute dissolution. The agitation is stopped when the solute does not decrease significantly, and then is allowed to stand for some time. The liquid supernatant is sampled and its composition is analyzed to obtain the solubility of the system at a certain temperature. The specific process was as follows: A volume of 100 mL solvent was added into the crystallizer and the thermostatic water bath turned on. After the temperature of the water bath was constant, excessive PYX crystals were added to the crystallizer. There was constant temperature stirring for a while, the stirring was then stopped and stood at a constant temperature after the solid and liquid tended to balance. A certain quality of supernatant solution was absorbed into a weighing bottle of known quality, and dried at 80 °C for 48 h to ensure that the solvent was completely volatilized. After the weighing bottle was cooled to room temperature, the mass of the weighing bottle and sample were weighed. The total mass minus the known mass of the measuring bottle, the solute mass, and the solubility were obtained. The undissolved crystals in the crystallizer were dried and characterized to ensure that no crystal transformation occurred before and after measurement. Each experiment was repeated three times, and the average was taken. The molar fraction solubility xA of the solute can be calculated by Equation (12).

(12) $$X_{A}=\frac{m_{A}{/M}_{A}}{m_{A}{/M}_{A}{+m}_{B}{/M}_{B}{+m}_{C}{/M}_{C}}$$

where x_A_ is the mole fraction. m_A_, m_B_, m_C_ represent the mass of the solute, solvent B and solvent C. M_A_, M_B_, and M_C_ represent the molecular weight of the solute and two solvents, respectively.

### 3.3. Characterizations

The morphology was directly observed by the field emission scanning electron microscope (FESEM, Hitachi S-4800, Hitachi Ltd., Tokyo, Japan). The chemical structure of the crystals was characterized by infrared spectrometer (IR, Bruker Tensor 27, Brucker Ltd., Billerica, MA, USA). The chemical purity was obtained by liquid chromatograph (Waters ZQ 2000, Waters Ltd., Milford, MA, USA). An Agilent HC C18 column was used, the wavelength was 254nm, and the column temperature was 30 °C. The mobile phase was acetonitrile/(PH = 3 phosphoric acid aqueous solution) = 50/50, and the flow rate was 1.0 mL/min. Thermal analysis was determined by differential scanning calorimetric (DSC) and thermogravimetric (TG) analyses (Netzsch STA 449 F3). Around 3 mg sample was heated in the argon (50 mL/min) from room temperature to 500 °C with different heating rates of 5 °C/min, 10 °C/min, 15 °C/min, and 20 °C/min. The crucible was a 70 μL alumina crucible with an outer diameter of 6 mm and a height of 4.5 mm. The friction sensitivity was tested according to the GJB-772A-97 method 602.1: the explosion probability method. The test conditions were as follows: pendulum mass was 1.5 kg, pendulum Angle was 90°, pressure gauge was 3.92 MPa, sample amount was 20 mg, ambient temperature was 20 °C, and humidity was 60%. Impact sensitivity was tested according to GJB-772A-97 method 601.1 explosion probability method, and the test conditions were as follows: weight of drop hammer was 10 kg, height was 25 cm, sample amount was 50 mg, test ambient temperature was 20 °C, the humidity was 60%.

## 4. Conclusions

The crystal morphology, surface smoothness, particle size distribution, and internal and external defects of explosives will affect the process, sensitivity, mechanical strength, detonation, and other properties of composited explosives. The crystal morphology of PYX is mostly needle crystals with low packing density and poor dispersion, which is difficult to meet the requirements of raw materials used in composited explosives. Therefore, it is of great practical significance to recrystallize PYX with fewer internal defects, smaller aspect ratio, higher roundness, and smoother surface. The solvent–antisolvent method has the advantages of low energy consumption, it is simple process, it has low cost, and is widely used in the crystallization of crystals. In this paper, the recrystallization of PYX in different solvent systems was studied. The aspect ratio of recrystallized sample was decreased from 3.47 to 1.19, the roundness value was improved from 0.47 to 0.86, and the median diameter was 2.993. The impact sensitivity decreased by 28%, and the thermal decomposition temperature was 5 °C later than raw PYX. The particle size and morphology can be controlled by changing the recrystallization process and thus meets the need for the grain size distribution of composited explosives. Recrystallization can improve the thermal stability of PYX and meet the application of heat-resistant composited explosives in high-temperature environments. In conclusion, it is of great significance to repair the crystal shape of PYX needle explosives for application in weapons.

## Figures and Tables

**Table 1:** Apelblat equation regression parameters of PYX in three solvents.

Solvent	A	B	C	100 RD	100 RMSD	R^2^
DMSO	−152.286	4195.838	24.397	0.127	2.0619	0.99739
NMP	−163.224	6521.484	25.328	0.0662	0.4758	0.99936
DMF	−0.27942	113.707	0.425	0.0073	0.263	0.99379

**Table 2:** Van’t Hoff equation regression parameters of PYX in three solvents.

Solvent	a	b	100 RD	100 RMSD	R^2^
DMSO	−3489.233	12.472	0.145	2.4908	0.99617
NMP	−1456.777	7.820	0.0140	1.5207	0.99188
DMF	−20.159	2.590	0.0195	0.058	0.98289

**Table 3:** Theoretical and experimental values of the solubility of PYX at different temperatures.

Solvent	T/K	lnx_A,exp_	Van’t Hoff Equation		Apelblat Equation	
			lnx_A,cal_	100 RD	lnx_A,cal_	100 RD
DMSO	298.15	0.76733	0.769055	−0.22483	0.791177	−3.10775
	303.15	0.9986	0.962078	3.657368	0.964812	3.383509
	308.15	1.13119	1.148836	−1.55995	1.139343	−0.7207
	313.15	1.3265	1.329631	−0.236	1.31462	0.895563
	318.15	1.48983	1.504742	−1.00095	1.490511	−0.04572
	323.15	1.63019	1.674435	−2.71413	1.666892	−2.25138
	328.15	1.85015	1.838957	0.604968	1.843649	0.351364
	333.15	2.03035	1.998541	1.566696	2.02068	0.476251
NMP	298.15	2.953	2.933946	0.011648	2.957894	−0.14946
	303.15	3.02098	3.014534	0.213373	3.01836	0.086733
	308.15	3.0842	3.092507	−0.26934	3.083642	0.018096
	313.15	3.14229	3.16799	−0.81787	3.153401	−0.35361
	318.15	3.22672	3.2411	−0.44566	3.227324	−0.01872
	323.15	3.30399	3.311948	−0.24086	3.305119	−0.03416
	328.15	3.3887	3.380637	0.237941	3.386514	0.064519
	333.15	3.4663	3.447264	0.549174	3.471258	−0.14303
DMF	298.15	2.52313	2.522386	0.029472	2.523434	−0.01204
	303.15	2.52408	2.523502	0.022917	2.524212	−0.00522
	308.15	2.52502	2.524581	0.017403	2.525078	−0.00231
	313.15	2.52596	2.525625	0.013258	2.526027	−0.00266
	318.15	2.5269	2.526637	0.010416	2.527053	−0.00605
	323.15	2.52783	2.527617	0.008418	2.52815	−0.01267
	328.15	2.52877	2.528568	0.007999	2.529314	−0.02152
	333.15	2.53065	2.52949	0.045849	2.530541	0.00432

## Data Availability

The data presented in this study are available on request from the corresponding author.

## Sample Availability

Samples of the compounds are available from the authors.
